# Experimental and computational validation of models of fluorescent and luminescent reporter genes in bacteria

**DOI:** 10.1186/1752-0509-4-55

**Published:** 2010-04-29

**Authors:** Hidde de Jong, Caroline Ranquet, Delphine Ropers, Corinne Pinel, Johannes Geiselmann

**Affiliations:** 1 Institut Jean Roget, LAPM, UMR5163, Campus Santé, Université Joseph Fourier, Domaine de la Merci, 38700 La Tronche, France; 2 INRIA Grenoble - Rhône-Alpes, 655 Av. de l'Europe, Montbonnot, 38334 St Ismier Cedex, France

## Abstract

**Background:**

Fluorescent and luminescent reporter genes have become popular tools for the real-time monitoring of gene expression in living cells. However, mathematical models are necessary for extracting biologically meaningful quantities from the primary data.

**Results:**

We present a rigorous method for deriving relative protein synthesis rates (mRNA concentrations) and protein concentrations by means of kinetic models of gene expression. We experimentally and computationally validate this approach in the case of the protein Fis, a global regulator of transcription in *Escherichia coli*. We show that the mRNA and protein concentration profiles predicted from the models agree quite well with direct measurements obtained by Northern and Western blots, respectively. Moreover, we present computational procedures for taking into account systematic biases like the folding time of the fluorescent reporter protein and differences in the half-lives of reporter and host gene products. The results show that large differences in protein half-lives, more than mRNA half-lives, may be critical for the interpretation of reporter gene data in the analysis of the dynamics of regulatory systems.

**Conclusions:**

The paper contributes to the development of sound methods for the interpretation of reporter gene data, notably in the context of the reconstruction and validation of models of regulatory networks. The results have wide applicability for the analysis of gene expression in bacteria and may be extended to higher organisms.

## Background

Fluorescent and luminescent reporter genes are popular tools for quantifying gene expression. The underlying principle of the technology is to fuse the promoter region and possibly (part of) the coding region of a gene of interest to a reporter gene. The reporter gene can be expressed from a (low-copy) plasmid or integrated at a suitable location in the host chromosome. The expression of the reporter gene generates a visible signal (fluorescence or luminescence) that is easy to capture and reflects the expression of the gene of interest (*e.g*., [1-5]).

The use of reporter genes allows real-time monitoring of gene expression, both at the level of individual cells and cell populations. By means of single-cell fluorescence and luminescence microscopy, fluctuations in gene expression due to internal and external noise can be measured. This has led to new insights into the ways cells both reduce and exploit these fluctuations (see [6-8] for reviews). Automated microplate readers measure gene expression of cell populations rather than individual cells. The lower resolution is compensated by a substantially higher throughput, as several dozens of genes can be monitored in parallel, at a much higher precision and sampling density than is currently possible by means of, *e.g*., DNA microarrays. The availability of libraries of fluorescent and luminescent reporter gene plasmids has further contributed to the potential of the technology [9,10].

Several examples of the real-time quantification of reporter gene expression on the population level have appeared in the literature in recent years. These examples include the monitoring of gene expression in the lysis-lysogeny decision in bacteriophage *λ *[11], the oxidative stress [12] and DNA damage response [13,14] in *E. coli*, the thermal induction of virulence factors in *Y. pestis *[15], the mapping of the regulatory region of the *lac *operon [16], and the dynamics of synthetic genetic regulatory networks [17]. In a typical microplate experiment, 96 cultures are followed in parallel, over several hours. This results in large amounts of data, of the order of 10,000-100,000 measurements of absorbance and fluorescence and luminescence intensities per experiment. In order to meaningfully interpret these data, we need to assess what exactly reporter gene measurements can teach us about the actual processes going on in the cell. Mathematical models have been shown critical for inferring biologically relevant quantities from reporter gene data (*e.g*., [13,18-23]). Most approaches present ways to infer the promoter activity from the primary data. By genetic construction, the measured promoter activity of a reporter gene carries over to any host gene that is under the control of the same promoter. Some studies have inferred the concentration profile of a transcription factor controlling the promoter by means of a known or hypothesized kinetic expression for the mechanism by which the transcription factor controls the promoter (see [13,20] for good examples). Another approach is to reconstruct (relative) measures of the reporter mRNA and protein concentrations from the data and use these as estimates of the corresponding products of the host gene. This approach is intuitively attractive, as it allows a straightforward read-out of the expression of any gene whose regulatory sequences are cloned into a reporter construct. However, it poses the question of the accuracy of the estimates, because the kinetics of host and reporter gene expression may be different. The aim of this paper is to systematically investigate this question by means of a combination of models and experiments. Our specific contributions are the experimental validation of the approach by comparing the quantities reconstructed from the reporter gene data with direct measurements of the accumulation of mRNA and protein, obtained by Northern and Western blots, respectively. Moreover, we use the models to pinpoint potential systematic biases arising from the folding time of fluorescent reporter proteins, and from differences in the half-lives of the products of host and reporter genes. This allows us to correct for the resulting systematic errors in the measurements and obtain a more accurate estimate of synthesis rates and concentrations of the host protein.

To illustrate the interest of this approach for the analysis of gene expression in bacteria, we have constructed fluorescent and luminescent reporter systems of the gene *fis *of *E. coli*. More specifically, we have cloned the *fis *promoter into plasmids containing either a gene coding for a Green Fluorescent Protein (GFP), or an operon encoding the enzymes of a light-producing reaction catalyzed by bacterial luciferase. The *E. coli *host gene codes for the protein Fis, a global regulator of transcription that plays a central role in, among other things, the control of metabolism and the coupling of the DNA topology to cellular physiology [24]. The expression pattern of *fis *has been thoroughly investigated before: *fis *expression is induced after a glucose upshift and decreases subsequently when the bacteria enter the exponential phase of growth [25-27]. It thus serves as an ideal example of a transient response in bacterial gene expression. A first interesting finding is that the relative mRNA and protein concentrations obtained from the reporter gene data are in good overall correspondence with the Northern and Western blot measurements, respectively. This suggests that the use of fluorescent and luminescent reporter genes in combination with automated microplate readers may yield reasonably accurate estimates of the expression profile of the products of the host gene. Second, we show that corrections for systematic biases due to differences in the half-lives of reporter and host mRNAs have mostly negligible effects, whereas corrections for differences in the half-lives of reporter and host proteins further improve the agreement between the inferred Fis concentration profiles and the Western blots. This conclusion, strengthened by simulation studies, suggests that the latter differences may need to be taken into account when using reporter gene data for the reconstruction of regulatory networks. Our work has wide applicability for the interpretation of measurements of gene expression in microorganisms.

## Methods

### Plasmids and strains

*Escherichia coli *strain BW25113 was used as a wild-type strain [28]. The plasmids used in this study are listed in Section S6 of the Additional file 1. The *gfp*- and *lux*-containing plasmids (pZEgfp and pSBluc) are derivatives of plasmids pZE1RM [17] and pSB377 [29], respectively, with a modified sequence of the multiple cloning site. The sequence between the end of the multiple cloning site (*EcoR *I) and the start codon (ATG) of *luxC *and *gfp *is: gaattcCCCG GGTAATTCAG GCCTGGAGGA TACGTatg and gaattcCCCG GGTAATTCAT TAAAGAGGAG AAAGGTACCG Catg, respectively. We have amplified the promoter region of *fis *by PCR from genomic DNA of *E. coli*, with oligonucleotides Fis1 and Fis2 (Fis1: ATCGCTCGAG GTGACGCGG, Fis2: TACG GAATTC GAGTTAAGAA ATGACCATAC TGTGA). Oligonucleotide Fis1 contains an *Xho*I restriction site, and oligonucleotide Fis2 an *EcoR*I restriction site, which allows cloning of the amplified DNA between these two sites on plasmids pSBluc and pZEgfp. The resulting plasmids are called pSB-fislux and pZE-fisgfp, respectively. Plasmids were verified by sequencing. They possess a *colE1 *origin of replication, are present at about twenty copies per cell, and do not affect bacterial growth (data not shown).

### Experimental conditions

Glycerol stocks, stored at -80°C, of strains BW25113 [28] carrying (or not) a plasmid-encoded reporter gene were grown overnight (≈ 15 h) at 37°C, with shaking at 200 rpm, in M9 minimal medium [30] supplemented with 0.3% glucose. For plasmid-carrying strains, the growth medium was supplemented with 100 *μ*g·ml^-1^ampicillin. The overnight culture was diluted 20-fold into the same, fresh medium. After 4 hours of growth the culture medium was changed by centrifugation and resuspension in M9 without glucose. The volume was adjusted in order to obtain an OD_600 _of 0.2. The bacteria were incubated without nutrients at 37°C for an additional 15 hours. Abruptly limiting the glucose availability in this fashion assures that the bacteria are in a defined physiological state at the beginning of the experiment. For the upshift experiments, 50 *μ*l of these growth-arrested cultures were added to 100 *μ*l of prewarmed M9 medium, containing glucose at a final concentration of 0.15%, and grown in a microtiter plate (≈ 12 h) at 37°C. The microplates were agitated at regular intervals during growth in the Fusion microplate reader (Perkin Elmer). During a typical experimental run we acquire about 100 readings each of absorbance, luminescence, and fluorescence. Fluorescence excitation was at 485 nm and emission was monitored at 520 nm. Absorbance measurements used a 600 nm filter.

### Data analysis

The absorbance, luminescence, and fluorescence data were fitted with regression splines, using the Spline toolbox of Matlab (Mathworks). In the absence of a specific parametric model of the data, regression splines provide a flexible, non-parametric modeling framework that allows estimation of the underlying trend in the absorbance and light intensity. In particular, we have used cubic B-splines [31] in combination with the generalized cross-validation (GCV) criterion for determining the number and the placement of the knots [32]. The optimal spline fit is the one minimizing GCV, that is, minimizing the residual sum of squares subject to a penalty term increasing with the number of knots (Section S2 of the Additional file 1). In order to find an estimate of the minimizer of GCV, and therefore of the 'best' choice of knots, we have followed a simple, stepwise knot selection schema [33]. The actual computation of the regression spline from a knot sequence is carried out by the Matlab function spap2.

A major advantage of the use of splines is that they greatly facilitate the computation of derived quantities from the primary data. Since splines are piecewise-polynomial functions, standard arithmetic operations, as well as differentiation and integration operations, can be carried out analytically [31]. This is more efficient and leads to more precise results than the use of numerical approximations. The latter cannot be completely avoided though, as some of the expressions that need to be evaluated for the computation of the host protein synthesis rate and host protein concentration involve functions that are not splines (Section S4 of the Additional file 1). In this case the integrals are computed by means of the Matlab function quad.

For each of the derived quantities, we computed 95% confidence bands using a standard bootstrap method. In particular, we have followed the residual resampling scheme [34], which constructs bootstrap data sets by repeatedly resampling the residuals of the optimal spline fit (Section S5 of the Additional file 1). For each of the 200 bootstrap data sets generated, we computed the synthesis rates and concentrations of the host and reporter proteins. From this empirically determined distribution, we obtained an estimate of the 95% confidence interval for the predicted values at evenly-spaced time-points, using so-called bootstrap percentiles [34]. The confidence bands shown in the figures in the text have been obtained by connecting the estimates of the point-wise confidence intervals.

### Background correction

For each type of measurement, an appropriate procedure for background correction has been developed. The absorbance background is detected by performing measurements on wells without bacteria, containing growth medium only. One would expect these background levels to be constant over time, which is confirmed by the actual measurements (data not shown). Denoting by *A*_*u *_the uncorrected absorbance and by *A*_*b *_the background absorbance, we define the corrected absorbance *A *as:(1)

The fluorescence background is determined by measuring the fluorescence of a strain carrying the promoterless vector pZEgfp. The background fluorescence is not constant, but rather varies with the population size due to the autofluorescence of bacterial cells. In this case, direct subtraction of the background readings from the uncorrected fluorescence intensity at each time-point *t *is not appropriate, as the size of the bacterial population generating the uncorrected signal is generally different from the size of the population generating the background signal.

We therefore first compute the average fluorescence intensity per cell for the uncorrected signal and the background signal. We denote by *B*(*t*) the absorbance of the strain carrying the promoterless vector, *A*(*t*) the absorbance of the strain with the functional reporter system, *I*_*u*_(*t*) the uncorrected fluorescence intensity, and *I*_*b*_(*t*) the background fluorescence intensity. The average fluorescence intensity per cell for the uncorrected and the background signal are then given by *I*_*u*_(*t*)/*A*(*t*) and *I*_*b*_(*t*)/*B*(*t*), respectively. We subtract the latter from the former to obtain the corrected average fluorescence intensity per cell, which we then multiply by the population size, as estimated by the absorbance, to obtain the corrected fluorescence intensity *I*(*t*):(2)

The background correction for the luminescent measurements could, in principle, be carried out in the same way as the one for the fluorescence measurements. However, as the luminescence background is quite low in practice, simple background subtraction is usually sufficient:(3)

### Western and Northern blot analysis

Equal quantities of protein were separated on 18% SDS-PAGE acrylamide gels and transferred onto nitrocellulose filters (Amersham Pharmacia). Filters were incubated with anti-Fis antibodies. Immunoblots were developed by using horseradish peroxidase-conjugated goat anti-rabbit antibody, followed by enhanced chemiluminescence (Amersham). The image of the blot acquired with a highly sensitive CCD camera and averaged for two minutes was quantified using the ImageJ software [35].

Total RNA was extracted from cells using the hot phenol procedure [36], or the Trizol procedure (Invitrogen). RNA samples were stored in DEPC water at -80°C until further use. The total RNA was loaded on a polyacrylamide (6% TBE-Urea, Invitrogen) or agarose gel (1%). After migration, the RNA was transferred to a Hybond-N membrane (Amersham Biosciences) and crosslinked with UV (1200 J). The membrane was prehybridized in Ultrahyb (Ambion) for 1 h at 42°C, followed by addition of radiolabeled oligonucleotide probe and hybridization overnight at 42°C. Membranes were washed twice with 2× SSC/0.1% SDS at room temperature followed by one wash with 2× SSC/0.1% SDS at 42°C for 2 min. Oligonucleotide probes were labelled by polynucleotide kinase according to manufacturer protocols (Fermentas) using [32P] ATP (6000 Ci/mmole; Perkin-Elmer). Probes were purified over mini quick spin columns (Roche) prior to use. Membranes were exposed on a phosphor screen, the screen revealed on a FLA-8000 (Fujifilm), and the image of the film quantified using ImageJ. The sequences of the probes used are listed in Section S6 of the Additional file 1.

### Measurement of degradation constants

To determine the degradation constant *γ*_*q *_of the GFP reporter of *fis*, we grew a bacterial culture under the experimental conditions described above to exponential phase and added chloramphenicol to 100 *μ*g/ml. The fluorescence data obtained after growth arrest were fitted by an exponential to yield the degradation constant. A similar procedure was followed for the luciferase reporter. A value for the degradation constant *γ*_*p *_of Fis was obtained by growing cells to the same growth stage and treating them with spectinomycine (100 *μ*g/ml). 1 ml samples were removed every hour during 5 h and treated as described in the section on Western blot analysis. An exponential fit gave the value of *γ*_*p*_.

To determine the degradation constant *γ*_*n *_of the reporter mRNA, strains BW25113 containing either plasmid pZACR105 (*gfp*) or pZACR101 (*lux*) were used (Section S6 of the Additional file 1). In these plasmids, the *gfp *gene or the *lux *operon are cloned downstream of the PLtetO-1 promoter that is controlled by the TetR repressor ([37]; Ranquet *et al*., in preparation). Derepression of the promoter is achieved by adding anhydrotetracycline (aTc). The strains were grown at 37°C to mid-log phase in LB medium, and aTc (500 ng/ml final) was added for 30 min to induce transcription of *gfp *or *lux*. Rifampicine (150 g/ml final) was then added to stop transcription and samples were taken every minute during 10 min. mRNA was isolated and detected as described in the section on Northern blot analysis. The degradation constant *γ*_*m *_of *fis *messages was determined by growing the strain BW25113 in LB to mid exponential phase, where Fis is the most abundant. Rifampicine was added and the mRNA was extracted as described above.

## Results

### Modeling reporter gene systems

In order to measure the expression of the gene *fis *in *E. coli*, we have constructed two reporter plasmids with identical backbones, including the antibiotic resistance gene and the origin of replication. The first contains the gfpmut3*-asv reporter gene, a variant of the gene coding for the Green Fluorescent Protein (GFP) from the jellyfish *Aequorea victoria *[38]. The second plasmid carries the *luxCDABE *operon from *Xenorhabdus luminescens*, encoding the enzymes of a light-producing pathway in this bacterium [39]. Because *fis *has its expression controlled at the transcriptional level [26,27,40], we prepared transcriptional fusions in which the promoter region of *fis *is fused to the *gfp *gene or the *lux *operon.

**Figure 1:**
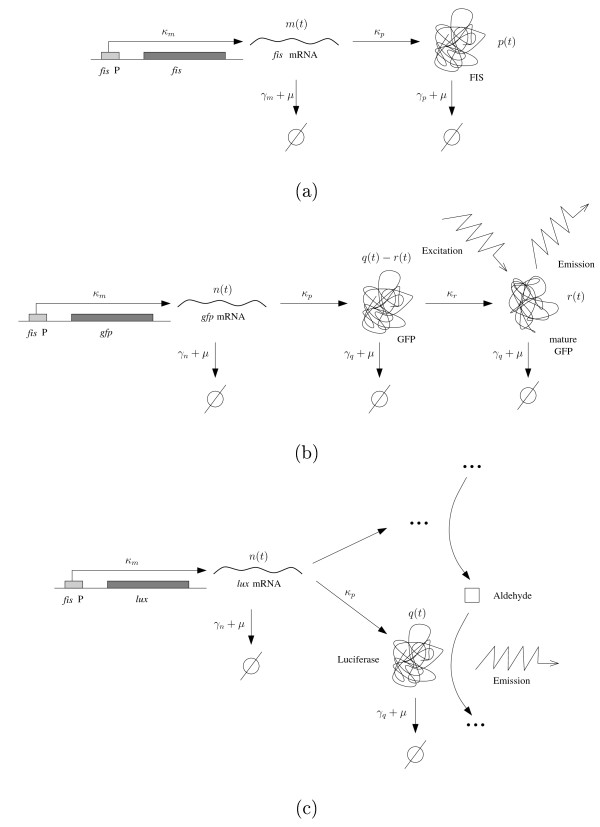
**Schematic representation of the expression of host and reporter genes**. (a) Expression of the gene *fis*, involving transcription, translation, and growth dilution and degradation of the gene products. (b) Expression of the *gfp *reporter gene, involving in addition to panel a a folding reaction. (c) Expression of the *lux *operon encoding luciferase and the enzymes producing the substrate of the luciferase-catalyzed reaction. The latter enzymes are not explicitly shown in the figure. The kinetic constants refer to equations (4)-(8).

Figure 1 summarizes the relationship between the expression of the host gene and the reporters.

Transcription of the gene *fis *gives rise to *fis *mRNA, which is subsequently translated into Fis protein. The synthesis of mRNA and protein is counterbalanced by growth dilution and degradation of the gene products. Together these processes determine the net accumulation of mRNA and protein in the cell. The expression of the *gfp *reporter gene follows roughly the same stages, with an important difference though. Fluorescent activity of GFP in response to light excitation depends on post-translational modifications, notably the folding of the protein to an appropriate conformation, including the autocatalytic formation of the chromophore [41]. This maturation process gives rise to an additional reaction step from GFP to active GFP (Figure 1). In the luminescent reporter gene system, light is not emitted in response to an excitatory signal, but as a by-product of an oxidation reaction. This reaction is catalyzed by the heterodimeric enzyme luciferase and requires a substrate, a long-chain aldehyde, which is synthesized by enzymes co-expressed with luciferase from the *lux *operon [39].

The expression of the host gene is modeled in the classical way [42-45], by means of differential equations describing the evolution of the cellular mRNA concentration *m*(*t*) and protein concentration *p*(*t*) as a function of time *t*:(4)

The mRNA synthesis rate in (4) is given by a maximum transcription rate *κ*_*m *_multiplied by the time-varying promoter activity *f*(*t*), a nonlinear function of time normalized to a value between 0 and 1. The mRNA synthesis rate is also called promoter activity. The mRNA decay rate is the sum of the growth dilution and degradation rates, accounted for by the term (*μ*(*t*) + *γ*_*m*_) *m*(*t*). Here, *μ*(*t*) denotes the growth rate as a function of time and *γ*_*m *_the degradation constant for *fis *mRNA. The protein synthesis rate in (5) is given by *κ*_*p*_*m*(*t*), where *κ*_*p *_is the translation rate constant. (The term promoter activity is sometimes also used for the protein synthesis rate, when models are used that lump together the transcription and translation steps (*e.g*., [13]).) The protein decay rate is again composed of a growth-dilution and degradation contribution, with *γ*_*p *_the degradation constant of protein Fis. The variables and constants used in (4)-(5) and below are summarized in Table 1.

**Table 1:** Variables and constants used in the models of the expression of the host and reporter genes.

	Concentration variables		
*m*(*t*)	host mRNA concentration [M]	*n*(*t*)	reporter mRNA concentration [M]
*p*(*t*)	host protein concentration [M]	*q*(*t*)	total reporter protein concentration [M]
		*r*(*t*)	active reporter protein concentration [M]

	Promoter activity and growth rate		

*f*(*t*)	promoter activity [dimensionless]	*μ*(*t*)	growth rate [min^-1^]

	Kinetic constants		

*κ*_*m*_	transcription rate constant [M min^1^]	*κ*_*p*_	translation rate constant [min^-1^]
		*κ*_*r*_	folding rate constant [min^-1^]
*γ*_*m*_	host mRNA degradation constant [min^-1^]	*γ*_*n*_	reporter mRNA degradation constant [min^-1^]
*γ*_*p*_	host protein degradation constant [min^-1^]	*γ*_*q*_	reporter protein degradation constant [min^-1^]

The same model is used for the transcription and translation steps of the *gfp *reporter gene, with the understanding that new variables *n*(*t*) and *q*(*t*) are introduced for the mRNA and protein concentrations of the reporter, respectively:(6)

In comparison with the model of *fis *expression, *γ*_*n *_and *γ*_*q *_denote the degradation constants for mRNA and protein, respectively. An additional differential equation accounts for the maturation of GFP:(8)

Here, *r*(*t*) stands for the concentration of active GFP, as compared to the total GFP concentration *q*(*t*), and *κ*_*r *_is the rate constant for the first-order folding reaction. *κ*_*r *_(*q*(*t*) - *r*(*t*)) thus represents the folding rate and we call ln 2/*κ*_*r *_the folding time of GFP. The model (6)-(8) can, with some variations, be found in other work [20-23,46].

Notice that a number of implicit assumptions underlie the above models of host and reporter gene expression. First, the promoter activity *κ*_*m *_*f *(*t*) characterizes the transcription of both the host and reporter genes, which is a direct consequence of the use of transcriptional fusions to measure *fis *expression. Second, we assume that the translation constant is the same for host and reporter gene expression. In the case of Fis this is justified by the fact that translation is not regulated [26,27,40]. In situations where this assumption is not valid, and post-transcriptional regulation occurs, translational fusions to the *gfp *reporter gene should be used. Third, the degradation constants of active and inactive GFP are assumed to be identical, which is reasonable in the absence of evidence to the contrary. Fourth, delays in transcription and translation are small with respect to the folding time and can safely be ignored here. Fifth, the growth characteristics of the wild-type and reporter strains are the same, an assumption that we have validated by comparing the growth rates of the two strains (data not shown).

The model of reporter gene expression was specifically developed for the case of GFP, but it can be adapted in a straightforward manner to the luminescent reporter gene system. Assuming that the substrates of the light-producing reaction are not rate limiting, the dynamics of the system is conveniently described by the temporal evolution of the luciferase concentration. We have verified this assumption for the aldehyde substrate and molecular oxygen, O_2_. The third substrate of the luciferase reaction, FMNH_2_, is directly related to the reducting power of the cell and can become rate-limiting in particular physiological situations, such as severe depletion of carbon sources. We observe a mild manifestation of this effect at the entry into stationary phase (see Discussion). However, during exponential growth, none of these substrates is rate-limiting. Equations (6) and (7) thus remain valid, where *n*(*t*) and *q*(*t*) now represent the *lux *mRNA and luciferase concentrations, respectively. Since the activity of luciferase does not require the analog of a folding reaction, we simply replace (8) by the equation:(9)

That is, the total luciferase concentration equals the active luciferase concentration (see [47] for a more detailed model of the luminescent reporter system).

### Measurements by means of reporter gene systems

We have grown *E. coli *strains carrying the reporter plasmids in parallel on a microplate, in M9 minimal medium, and at a constant temperature of 37°C. The basic experiment consisted in adding glucose to a growth-arrested culture, following the protocol described in the Methods section, and repeatedly measuring the absorbance at 600 nm, as well as fluorescence and luminescence intensities. The time-series data were fitted to cubic regression splines using a minimization criterion that balances goodness of fit and parsimony (Methods and Additional file 1). The resulting spline fits of the primary data were corrected for background levels of absorbance, fluorescence, and luminescence. The background measurements were carried out on wells containing growth medium without bacteria (absorbance background), and on wells with strains carrying a reporter plasmid lacking a promoter upstream of the reporter gene (fluorescence and luminescence background) (see Methods).

**Figure 2:**
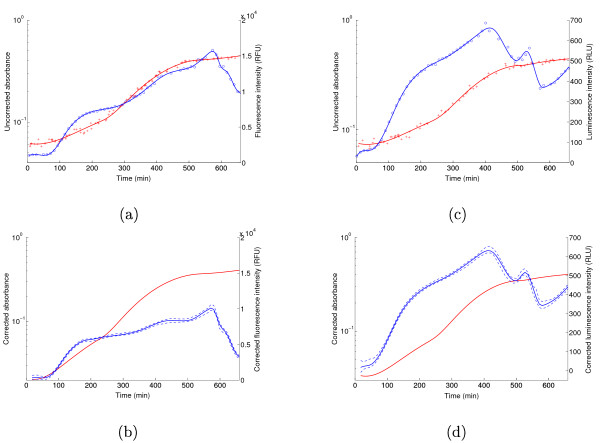
**Primary and corrected data**. (a) Absorbance and fluorescence intensity measured on a population of bacteria carrying the GFP reporter system of Fis. (b) Absorbance and fluorescence intensity corrected for background levels. (c)-(d) Idem for the luciferase reporter system of Fis. The measurements are represented by blue circles (fluorescence or luminescence) and red crosses (absorbance), and the spline fits are indicated by solid lines. The dashed lines delimit the 95% confidence bands.

The results obtained with the GFP and luciferase reporter plasmids of Fis are shown in Figure 2. At time zero, the growth-arrested bacterial cultures were diluted into fresh culture medium. The bacteria progressively reach the maximum growth rate in exponential phase, as can be seen with the logarithmic scale in the plots. The increase in fluorescence and luminescence levels accelerates after about one hour, but slows down later in exponential phase. When the culture enters stationary phase, the fluorescence and luminescence levels decrease due to the down-regulation of *fis*.

Panels b and d of Figure 2 show 95% confidence bands for the corrected absorbance and light intensity which were computed using the bootstrap method described in the Methods section. The confidence bands are tight, reflecting the high precision of the measurements, and the curves are reproducible (see Section S3 in the Additional file 1).

### Computation of reporter concentrations and synthesis rates

A central question for the interpretation of the primary data is how the latter can be related to the model variables. Let *I*(*t*) denote the corrected fluorescence or luminescence intensity over time, in relative fluorescence units (RFU) or relative luminescence units (RLU), respectively. Similarly, the dimensionless absorbance is denoted by *A*(*t*). The absorbance is proportional to the number of cells in a bacterial population. We have verified this assumption by counting the colony-forming units in parallel with the absorbance measurements (Section S1 in the Additional file 1). As a consequence, the ratio *I*(*t*)/*A*(*t*) represents the quantity of fluorescence or luminescence per cell as a function of time (*e.g*., [13,21]). The latter ratio can be related to the concentration of (active) reporter protein by making the reasonable assumption that the corrected fluorescence and luminescence intensities are proportional to the number of (active) GFP and luciferase molecules in the cells, respectively. We thus obtain:(10)

Since we do not know the proportionality constant in (10), we express concentrations in units RFU and RLU of the ratio *I*(*t*)/*A*(*t*). Notice that this provides a relative quantification of concentrations, as is usual in this kind of experiments. For most purposes, however, the relative concentrations are informative and robust measures of the dynamics of the system, for instance when we are interested in fold changes over the time-course of the experiment (see Discussion below). When this does not lead to ambiguities, we simply speak of concentrations instead of relative concentrations when we refer to variables with units RLU and RFU.

**Figure 3:**
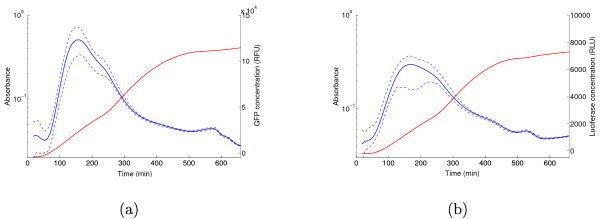
**Reporter concentrations**. (a) GFP concentration, computed by means of (10) from the data in Figure 2c. (b) Idem for the luciferase concentration. The dashed lines represent the 95% confidence bands.

Figure 3a-b shows how the reporter concentration, computed by means of (10), varies over time during exponential phase and after entry into stationary phase. The 95% confidence bands are obviously larger than for the light intensity and absorbance measurements in Figure 2, but they remain quite reasonable. Greater uncertainty at the beginning of the experiment is due to the larger relative errors when measuring small absorbance values. The expression profiles of GFP and luciferase are highly consistent in the sense that both reporter concentrations reach a peak around 150 min, and fall back to their initial value in stationary phase. Moreover, the fold change between the maximal concentration and the concentration reached at the entry into stationary phase is about 5 in both cases.

How is the protein synthesis rate determined from the primary data? Following (7), the translation rate is proportional to the mRNA concentration *n*(*t*) of the reporter gene with proportionality constant *κ*_*p*_:(11)

where the growth rate *μ*(*t*) can be computed from the absorbance profile in Figure 2 by means of the classical formula:(12)

The degradation constant *γ*_*q *_in (11) was measured as described in the Methods section. Its value is almost the same for the two reporters: 0.012 ± 0.001 min^-1 ^for GFP and 0.011 ± 0.001 min^-1 ^for luciferase, corresponding to a half-life of about 1 h (remember that the half-life equals ln 2/*γ*_*q*_.) In the case of luciferase we have *q*(*t*) = *r*(*t*), so that the total reporter concentration and its derivative can be directly determined from the primary data by means of (10). The total GFP concentration is not generally equal to the active GFP concentration, as explained above. However, for the time being, we will assume this equality to hold for GFP as well, before considering appropriate corrections at a later stage.

**Figure 4:**
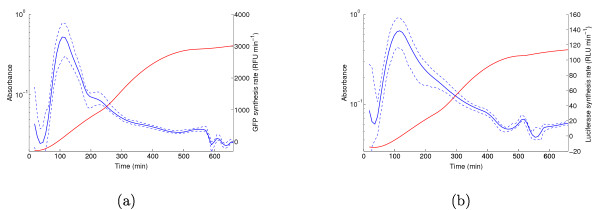
**Protein synthesis rates computed from reporter data**. (a) Protein synthesis rate computed by means of (11) from the GFP reporter data in Figure 2. (b) Idem for the luciferase reporter data.

In Figure 4 the protein synthesis rate computed from (11) is shown for the two reporter systems. The peak occurs two generation times after the glucose upshift, 30 min before the reporter concentration reaches its maximum. This is consistent with the fact that the protein synthesis rate is proportional to the mRNA concentration, whose peak should precede that of the protein concentration. However, contrary to what has been measured previously [25,26,48], Fis synthesis never stops completely during exponential growth of the bacterial culture. These results agree with recent microarray data showing that Fis actively regulates numerous genes in all growth phases [24], which is only possible if Fis is present in the cell. At first sight, one might suspect the variations around 500-600 min, at the entry into stationary phase, to be due to over-fitting. Closer inspection of the data, however, in particular when comparing the raw fluorescence and luminescence data of the experiment reported in Figure 2 with the data of its replicate in Section S4 of the Additional file 1, suggests that this is probably not the case. The rapid variations in the fluorescence and luminescence levels are small, but reproducible. In the case of the luminescence reporters, where they are most pronounced, we believe them to be partly due to metabolic changes occurring during glucose depletion, such as fluctuations in reducing power, which affect the activity of the light-producing reactions (see [39] and above).

The above analysis shows that, using (10)-(12), we are able to reconstruct the reporter concentration and the reporter synthesis rate (proportional to the mRNA concentration) from the primary data. The major question raised by this analysis is whether the reconstructed quantities for the reporter system reliably represent the corresponding quantities of the host system, that is, whether *n*(*t*) = *m*(*t*) and *q*(*t*) = *p*(*t*). As discussed above, this is *a priori *unlikely. Remember that in the case of GFP, we have neglected the maturation step, while the half-lives of the host and reporter mRNAs and proteins are generally different as well. On the other hand, if the expression profiles of the reporter genes turned out to be good approximations of those of the host gene, this would enormously simplify the analysis and interpretation of the data. We have therefore verified to which extent the reporter concentration and synthesis rate profiles computed from the reporter gene data deviate from direct measurements of the abundance of Fis protein and *fis *mRNA.

### Direct measurements of fis gene expression

**Figure 5:**
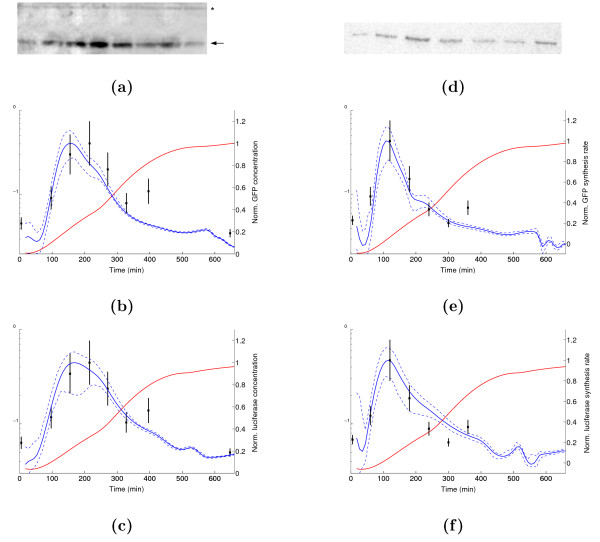
**Direct measurements of gene expression**. (a) Western blot of Fis at various stages of growth. Lanes 1 to 8 correspond to the times shown in b and c. The band corresponding to Fis is indicated by the arrow. A non-specific band recognized by the anti-Fis antibody (marked by an asterisk) has been used for normalization. (b) Correspondence between Western blot measurements (black squares) and GFP concentration. The dashed lines denote the 95% confidence bands. Both the reporter concentration and the Western blot values are normalized with respect to the peak in mid-exponential phase. (c) Idem for the luciferase concentration. (d) Northern blot of *fis *mRNA at various stages of growth. Lanes 1 to 7 correspond to the times shown in b and c. Equal amounts of total RNA were loaded in each lane. (e) Correspondence between Northern blot measurements (black squares) and GFP synthesis rate. Normalization is carried out in the same way as for the Western blot. (f) Idem for the luciferase synthesis rate.

We have measured the accumulation of Fis during growth on glucose by Western blots (see the Methods section for details). Figure 5a-c shows the projection of the Western blot measurements on the GFP and luciferase concentration profiles, both normalized with respect to the peak in mid-exponential phase. The profiles inferred from the reporter gene data using our models are in good agreement with the Western blot measurements. They reproduce the peak in mid-exponential phase, although the latter seems to slightly displaced to an earlier time-point (150 min vs 210 min). Notice, however, that the error bars of the Western blot measurements overlap with the confidence interval of the reporter gene profiles so that we cannot conclude with certainty that a discrepancy has occurred. The only significant deviation between the reporter gene and Western blot measurements occurs towards the end of exponential phase, where the curve computed from the reporter data clearly underestimates the Western blot quantification.

In a similar way, the synthesis rate of the GFP and luciferase reporters has been compared with Northern blot measurements at various stages of growth. Figure 5d-e shows the superposition of the Northern blot values and the synthesis rate profiles computed from the reporter gene data. The quantities have been normalized with respect to the value of the peak in exponential phase, as above. Following the definition of the reporter synthesis rate in (11), the normalized synthesis rate equals the normalized mRNA concentration. Again, there is a good overall correspondence between the profiles obtained from the reporter gene data and the direct measurements. Some significant deviations occur though, especially at the end of exponential phase (GFP data) and in mid-exponential phase (luciferase data).

We conclude from the agreement with direct measurements of Fis protein and *fis *mRNA that reporter genes are a reliable tool for tracking the shape of the expression profile of the host gene. It would be interesting to know if the local deviations that we also observe are due to the systematic biases identified above. In order to answer this question, we have developed computational procedures for correcting the profiles obtained from the reporter gene data for differences in half-life and for non-negligible folding times.

### Correction of systematic biases in computed protein and mRNA concentrations

In general, the half-lives of protein and mRNA will not be the same for Fis and its reporters, that is, *γ*_*m *_≠ *γ*_*n *_and *γ*_*p *_≠ *γ*_*q*_. This difference in half-life will cause the mRNA concentrations computed from the reporter data to deviate from the actual concentrations of *fis *mRNA. For example, the inferred concentration will be underestimated if *γ*_*n*_/*γ*_*m *_> 1, that is, if the *lux *or *gfp *message half-life is shorter than that of *fis*. Through the dependence of the protein synthesis rate on the mRNA concentration, this also affects the computed protein concentrations. The latter effect is modulated by possible differences in half-life of the host and reporter proteins. In particular, if *γ*_*q*_/γ_*p *_> 1, the error in the predicted mRNA concentration will be accentuated, whereas in the case of *γ*_*q*_/*γ*_*p *_< 1 it will be attenuated.

In order to quantify these systematic biases in our case, we experimentally determined the degradation constants of mRNA and protein of both Fis and its reporters, as described in the Methods section. The GFP and luciferase half-lives were measured to be about 1 h. For the degradation constant of Fis we found the value *γ*_*p *_= 0.0065 ± 0.0020 min^-1^, corresponding to a half-life of almost 2 h, twice as long as the half-life of the reporter protein. The difference is significant, as the 95% confidence intervals are disjoint: [0.89, 1.1] h and [0.96, 1.2] h, for GFP and luciferase, respectively, and [1.4, 2.6] h for Fis. The degradation constants *γ*_*n *_of *gfp *and *lux *mRNA were determined to be 0.30 ± 0.13 min^-1 ^and 0.33 ± 0.15 min^-1^, respectively, yielding half-lives of about 2 min. This is almost twice as long as the half-life for *fis *mRNA, equal to 1.23 min. Notice that the measurements are relatively imprecise, so that the 95% confidence intervals of the host and reporter mRNA half-lives are overlapping ([1.6, 4.1] min and [1.4, 4.1] min for *gfp *and *lux *mRNA, respectively, and [0.88, 2.1] min for *fis *mRNA). The measured kinetic constants and the errors on the measurements are summarized in Table 2.

**Table 2:** Measured values of the degradation constants in the models of fis, gfp and lux expression.

	GFP	Luciferase		Fis
*γ*_*q*_	0.012 (0.001) min^-1^	0.011 (0.001) min^-1^	*γ*_*p*_	0.0065 (0.0020) min^-1^
*γ*_*n*_	0.30 (0.13) min^-1^	0.33 (0.15) min^-1^	*γ*_*m*_	0.56 (0.23) min^-1^

Details of the experiments are given in the Methods section. Estimates of the 95% confidence intervals are shown between parentheses

The measurements of the kinetic constants allow the correction of systematic errors, using the models introduced above. As shown in Section S4 of the the Additional file 1, the Fis synthesis rate *κ*_*p*_*m*(*t*) can be numerically computed from the reporter synthesis rate *κ*_*p*_*n*(*t*), defined in (11), when the values of *γ*_*m *_and *γ*_*n *_are known. The only additional assumption needed is that, at the beginning of the experiment, the mRNA concentrations have attained their steady-state value, that is:(13)

This assumption is valid since in our experimental conditions the bacteria have been in stationary phase for more than 12 h before dilution into fresh growth medium.

**Figure 6:**
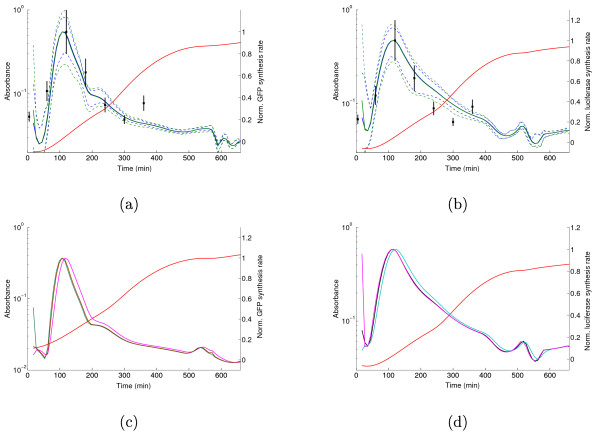
**Correction of protein synthesis rates for different mRNA half-lives**. (a) Original (blue line) and corrected (green line) GFP synthesis rate. The correction accounts for the systematic bias *γ*_*n*_/*γ*_*m *_= 0.54. Both profiles are normalized with respect to the peak in exponential phase. The 95% confidence bands are shown as dashed lines and the Northern blot measurements are taken from Figure 5. (b) Idem for luciferase, *γ*_*n*_/*γ*_*m *_= 0.59. (c) Robustness of computed protein synthesis rate (mRNA concentration) to systematic errors caused by differences in half-lives of *gfp *and *fis *mRNA. The figure shows the curves for *γ*_*n*_/*γ*_*m *_values equal to 0.25, 1, and 4. (d) Idem for *lux*.

The results of the correction of the systematic error in the reporter synthesis rate, and thus in the reporter mRNA concentration, are shown in Figure 6a-b. For the measured values of the ratio *γ*_*n*_/*γ*_*m*_, which equal 0.54 for *gfp *mRNA and 0.59 for *lux *mRNA, the difference in the normalized mRNA concentration profile is seen to be negligible, falling within the confidence band of the original predictions (corresponding to the case *γ*_*n*_/*γ*_*m *_= 1). This means that the local discrepancies between the Northern blot measurements in Figure 5 are not due to a difference between the half-lives of *fis *mRNA and that of *gfp *and *lux *mRNA.

This even holds for large half-life differences. As shown in Figure 6c-d, for values of *γ*_*n*_/*γ*_*m *_varying between 0.25 and 4, the predicted mRNA profile remains the same. Even when *γ*_*n*_/*γ*_*m *_is varied by 100-fold (see Additional file 1), the differences are quite moderate and the overall shape remains largely insensitive to this parameter. We observe that such large differences in half-life do not frequently occur in bacteria [49], contrary to what has been observed for yeast [50].

In a similar way, the protein concentration profile can be corrected for systematic errors due to differences in the degradation constants. The Fis concentration *p*(*t*) can be computed from the GFP or luciferase concentration *q*(*t*) when in addition to the values of *γ*_*m *_and *γ*_*n*_, those of *γ*_*p *_and *γ*_*q *_are known. As above, we need to make the further assumption that the system is initially at steady state, that is:(14)

The formulas required for the computation of *p*(*t*) are derived in Section S4 of the Additional file 1.

**Figure 7:**
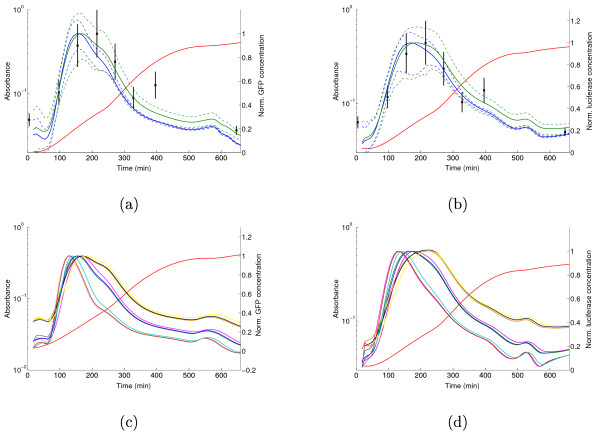
**Correction of reporter concentrations for different mRNA and protein half-lives**. (a) Original (blue line) and corrected (green line) GFP concentration profile. The correction accounts for the systematic bias *γ*_*n*_/*γ*_*m *_= 0.54 and *γ*_*q*_/*γ*_*p *_= 1.7. Both profiles are normalized with respect to the peak in mid-exponential phase. The 95% confidence bands are shown as dashed lines and the Western blot measurements are taken from Figure 5. (b) Idem for luciferase and Fis, *γ*_*n*_/*γ*_*m *_= 0.59 and *γ*_*q*_/*γ*_*p *_= 1.8. (c) Robustness of computed protein concentration to systematic errors caused by differences in half-lives of products of *gfp *and *fis*. The figure shows the curves for *γ*_*n*_/*γ*_*m *_and *γ*_*q*_/*γ*_*p *_values equal to 0.25, 1, and 4. The clearly separated curves correspond to different values of *γ*_*q*_/*γ*_*p*_. Within each set, the different ratios of the mRNA half-lives have very little effect. (d) Idem for *lux*.

The ratios of *γ*_*q*_/*γ*_*p *_were measured to be 1.8 (GFP) and 1.7 (luciferase). Figure 7a-b shows the effect on the predicted Fis profile. Contrary to the mRNA case, the corrections push the Fis profile locally outside the confidence band of the original, uncorrected profile. For both reporters, this leads to a better agreement with the Western blot measurements in the transition from exponential to stationary phase. The corrected profiles approach or capture the measurement at 400 min, which was missed by the original profile. This shows that the predictions can be improved by carrying out the corrections, but it also reveals that the better agreement comes at the price of slightly wider confidence bands.

Figure 7c-d shows that non-negligible differences may occur when the reporter concentration profiles are corrected for larger differences in half-life, although the profiles retain the same qualitative shape. In particular, the timing of the expression peak shifts according to the value of *γ*_*q*_/*γ*_*p*_. Notice that the expression profiles tend to cluster together around specific values of *γ*_*q*_/*γ*_*p*_, with minor variations within the clusters caused by differing values of *γ*_*n*_/*γ*_*m*_. The influence of the difference in mRNA stability is therefore negligible with respect to the difference in protein stability, confirming the results of Figure 6. In all of the above computations we have assumed that the folding time of GFP is negligible, implying that all GFP in the cell is active (*q*(*t*) = *r*(*t*)). This may lead to an underestimation of the amount of GFP in the cell. In order to correct for the effect of this bias, we can rewrite (8) to compute total GFP from active GFP:(15)

The maturation time of GFP was set to 25 min, as determined experimentally for the reporter used in this study (GFPmut3) [51], thus yielding a value *κ*_*r *_= 0.023 min^-1^. That is, it takes 25 min to convert half of a given pool of inactive GFP to its active form.

**Figure 8:**
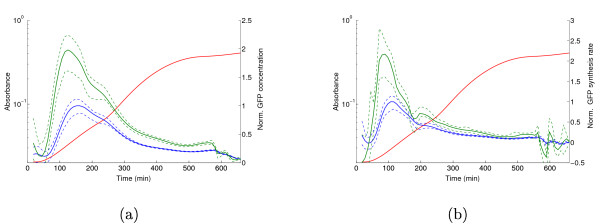
**Correction of GFP concentration and synthesis rate for folding time**. (a) Concentration profile of active GFP (blue line) and total GFP (green line). The latter profile has been corrected for the folding time of GFP. Both profiles are normalized with respect to the peak in mid-exponential phase of the active GFP concentration. (b) Concentration profile of *gfp *mRNA, before correction (blue line) and after correction (green line) for the folding time. Normalization is as in panel a.

Figure 8a shows the concentration profiles of both active and total GFP, normalized with respect to the peak in mid-exponential phase of the active GFP concentration. As expected, active GFP represents only a fraction of total GFP. However, the qualitative shape of the profiles remains essentially the same. Using the profile of *q*(*t*) instead of *r*(*t*) for computing the normalized reporter synthesis rate, and thus the normalized mRNA concentration, yields the same conclusion (Figure 8b). In both cases we see that the expression peak is slightly shifted to an earlier time-point. This is consistent with the fact that the maturation process introduces a delay in the availability of active GFP. The agreement of the computed profiles with the Western and Northern blot measurements is not improved by correcting for the folding time (result not shown).

We have also experimented with variants of GFP, in particular a rather slow folding RFP (Red Fluorescent Protein). In this case, there are considerable differences between the expression profiles obtained with luciferase (data not shown). The corrections and the corresponding confidence bands also become much larger. We conclude that a fast-folding reporter protein is essential for reliable real-time monitoring of gene expression.

## Discussion

Research in biology has moved from a descriptive science to considering biological processes as dynamical systems [52]. This systems biology approach relies on the analysis and interpretation of dynamical measurements and therefore calls for a precise mathematical treatment of quantitative time-series data of gene expression [13,18-23]. The present manuscript provides such an analysis by showing a way in which biologically relevant quantities, and their confidence intervals, can be rigorously computed from the primary data by means of kinetic models. In particular, in comparison with, for example [13,20], we infer relative mRNA and protein concentrations for a host gene using luminescent or fluorescent reporter systems under the control of the same promoter as the host gene. We extend previous work by explicitly stating and experimentally verifying the validity of the assumptions that underlie this procedure. We notably assess the effect on the model predictions of uncertain values for some of the parameters that are difficult or time-consuming to measure (such as the protein or mRNA half-lives). When such values are available, the computational procedures we provide can be used for correcting systematic errors due to differences in degradation constants.

A first conclusion from our study is that the expression profiles computed from the fluorescence and luminescence data are generally in good agreement with the Northern and Western blots (Figure 5). This is remarkable considering the fact that the measurements were obtained with completely different experimental methods and the comparison only involves normalization with respect to a maximum value, *i.e*., uses no freely adjustable parameters. It implies that when the half-lives of the host-gene products are unknown, we can still obtain a result that preserves the qualitative shape of the expression profile. As long as the systematic biases in the reporter systems remain limited, that is, a rapid folding time of the fluorescent reporter and similar degradation constants of host and reporter gene products, the expression profiles obtained are accurate. This is illustrated by the results for the gene *fis *coding for a global regulator in *E. coli*.

If the systematic biases are too large to be ignored, corrections for the resulting errors need to be carried out. Our results show that a difference in mRNA half-life does not significantly contribute to these deviations. As a consequence, knowing the order of magnitude of the mRNA half-life of the host gene is already sufficient for reliably calculating the expression profile. The insensitivity of the expression profile to changes in mRNA half-life does not hold for protein half-life. Variations in this parameter maintain the overall shape of the expression profile, but affect the normalized concentration levels and the timing of the peak (Figure 7). In particular, the simulation studies reveal that the longer the half-life of the host protein as compared to that of the reporter, the more the actual expression profile of the host gene is delayed. This effect has to be kept in mind when trying to reconstruct or validate models of regulatory networks based on reporter gene data [42,53-56]. It should notably be taken into account when attempting to infer network connections based solely on mRNA measurements, as in a typical microarray experiment. The effect of a particular protein will occur later than the transcription of its gene and the time delay depends on the protein half-life.

All computations have been carried out under the assumption that the mRNA half-life does not change in the course of the experiment. This assumption is certainly valid during exponential growth, but may fail during growth transitions or in situations where the mRNA half-life is regulated. Indeed, our data show a systematic deviation between the calculated and measured quantities of mRNA and protein at the entry into stationary phase that is partly unaccounted for, even after applying the above corrections. The mRNA and protein half-lives have been measured during exponential phase. Due to technical difficulties, we were unable to measure these parameters in stationary phase. It is conceivable that the mRNA half-life of *fis *increases at the transition to stationary phase. If this were the case, the actual mRNA and protein concentrations would be higher than the ones computed from the reporter gene measurements. This effect could indeed explain the remaining discrepancies between prediction and measurement in Figure 6. The analysis also confirms that the derived quantities, relative protein concentrations and synthesis rates (mRNA concentrations), are largely independent of the physical characteristics of the reporter gene system (Figures 3 and 4). This is quite remarkable given the vastly different physical properties of our two reporter systems. It is true that, in our data, we see some minor differences between the two reporter systems at the entry into stationary phase, notably visible in the protein synthesis rates (Figure 4). As explained in the Results section, these are most likely due to transient fluctuations of the reduction potential of the cell at the entry into stationary phase [57]. This difference must be kept in mind when interpreting reporter gene data and we recommend to always use two different reporter systems in parallel in order to separate gene expression from other effects. Identical profiles derived from the two reporter systems have a good chance to faithfully represent the true expression pattern of the host gene.

Finally, we note that the approach described in this paper yields relative rather than absolute measures of gene expression. As a consequence, the validation of the approach by means of Northern and Western blots concerns the comparison of relative values. In order to obtain an absolute quantification of protein concentrations, the proportionality constant in (10) needs to be determined by relating the fluorescence and luminescence intensity units to the number of (active) molecules, and the absorbance units to the number of (viable) cells. In addition, for an absolute quantification of mRNA concentrations the synthesis constant *κ*_*p *_needs to be measured. The techniques for doing this are time-consuming and error-prone, although novel approaches developed in the context of single-cell measurements may improve the absolute quantification of gene products (*e.g*., [58-60]). The calibration of the approach to obtain reliable absolute measures is an interesting perspective for further research. However, for many purposes in systems biology the determination of relative measures is sufficient, and our approach provides a speed-up and solid foundation for achieving this.

## Conclusions

Research in biology has made the transition from a more or less intuitive understanding of the system to a quantitative, formal description. This systems biology approach crucially depends on the availability of reliable, quantitative data. Data acquisition techniques have enormously progressed in the past decade, but require sound and general methods for analyzing these data. The current manuscript contributes to the development of such methods and forms the basis for future analyses of the dynamics of regulatory systems. The present formalism is geared towards bacterial expression. However, small modifications of the method will allow to include additional reaction steps inherent in eukaryotic gene expression, such as splicing and nuclear export.

## Authors' contributions

All authors made substantial contributions to the work presented in the manuscript. CR, DR and CP constructed the reporter strains. CR and CP carried out most of the experiments reported in the manuscript. DR also contributed to the analysis of the data and the development of the model. HdJ and JG conceived the study, developed the models, carried out some of the experiments, analyzed the data, and wrote the publication. All authors have read and approved the final manuscript.

## Supplementary Material

Additional file 1 Supplemental Material of Experimental and Computational Validation of Models of Fluorescent and Luminescent Reporter Genes in Bacteria. Click here for file
