# Supplementary material for: Experimental and computational validation of models of fluorescent and luminescent reporter genes in bacteria
Source: BMC Syst Biol. 2010 Apr 29;4:55. doi: 10.1186/1752-0509-4-55 (PMC2877006; doi:10.1186/1752-0509-4-55)
Supplement: Additional file 1 — Supplemental Material of Experimental and Computational Validation of Models of Fluorescent and Luminescent Reporter Genes in Bacteria. [file 1752-0509-4-55-S1.PDF]

Supplemental Material for "Experimental and computational validation of models of fluorescent and luminescent reporter genes in bacteria"

Hidde de Jong[2] Caroline Ranquet[1,2] Delphine Ropers[2]
Corinne Pinel[1,2] Johannes Geiselmann[1,2]
[1]Laboratoire Adaptation et Pathogénie des Microorganismes (CNRS UMR 5163)
Institut Jean Roget, Campus Santé, Université Joseph Fourier
Domaine de la Merci, 38700 La Tronche, France
[2]Institut National de Recherche en Informatique et en Automatique (INRIA)
Research center Grenoble – Rhône-Alpes
655 avenue de l'Europe, Montbonnot, 38334 Saint Ismier Cedex, France

April 11, 2010

S1 Absorbance measurements and colony-forming units

In order to investigate the assumption that the measured absorbance level is proportional to the number of bacteria, we took 20 μl and 80 μl samples at 7 different time-points during normal growth under the conditions described in Section 3.2 of the main text. The samples were diluted $50 \times 25 \times 25 = 31,250$ times and spread on an Agar plate. We counted the number of colonies for each sample, and calculated the corresponding number of colony-forming units (cfu) per ml. The results were plotted against the measured absorbance level (after background correction) at the time-points at which the samples were taken, as shown in Figure 9. The absorbance is indeed observed to be approximately proportional to the number of cfu per ml.

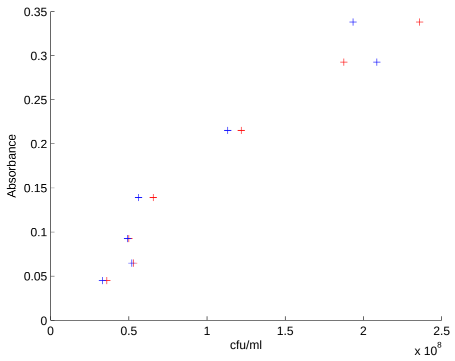

Figure 9: Plot of correlation between measured absorbance level and number of colony-forming units for samples taken at different time-points during growth. The results of two different experiments are shown, with sample sizes of 20 μl (in red) and 80 μl (in blue).

S2 Regression spline smoothing using generalized cross-validation (GCV)

We follow the presentation in (1). Suppose that measurements z_j have been made at time-points t_j, $j \in \{1, \ldots, n\}$, such that

$$z_j = f(t_j) + \epsilon_j, \ \epsilon_j \sim \text{i.i.d. } N(0, \sigma^2). \tag{S1}$$

f is a function to be recovered from the data, *e.g.*, in our case the (uncorrected) absorbance A_u or (uncorrected) fluorescence I_u (see Section 2.4 of main text). Suppose that f can be described by a cubic B-spline with parameters θ, consisting of the knot locations and the spline coefficients. The goal is to estimate f by a regression spline $\hat{f}$ with parameters $\hat{\theta}$. Various approaches can be followed to find the 'best' such estimator (2; 3). According to the generalized cross-validation (GCV) criterion, the optimal spline fit is given by the parameter vector $\hat{\theta}$ which minimizes

$$GCV(\hat{\theta}) = \frac{1}{n} \frac{\sum_{j=1}^{n} \left(z(t_j) - \hat{f}(t_j) \right)^2}{(1 - d(m)/n)^2}, \tag{S2}$$

where $d(m)$ is an increasing function of the number of knots m. We take $d(m) = 3m + 1$. In order to find $\hat{\theta}$ for which $GCV(\hat{\theta})$ is minimal, a variety of optimization algorithms can be used. A simple stepwise knot selection algorithm called Forward Addition, described in (1), was shown to give good results on our data.

S3 Reproducibility of measurements

The results presented in the main text demonstate that we can measure relative reporter concentrations and synthesis rates (proportional to mRNA concentrations) with high precision. However, an important criterium for the biological validity of the results is not only the quality of the data from one time-series measurement, but also the reproducibility of the results. Figure 10 compares the results in the main text with those from a replicate experiment.

A comparison of the expression profiles on all levels – measured fluorescence and luminescence intensities, reporter concentration, and reporter synthesis rate – demonstrates good reproducibility of the results. The confidence intervals confirm an obvious visual agreement: the replicate curves are mostly included in the confidence interval of the original time-series (Figure 10).

S4 Correction of systematic errors

We describe how, based on the models in Section 3.1 of the main text and measured values of the kinetic constants, the expression profiles inferred from the fluorescent and luminescent data can be corrected for systematic errors due to differences in the half-lives of the products of the reporter and the host gene. To make this section self-contained, we first repeat the equations of the model for the expression of the host gene:

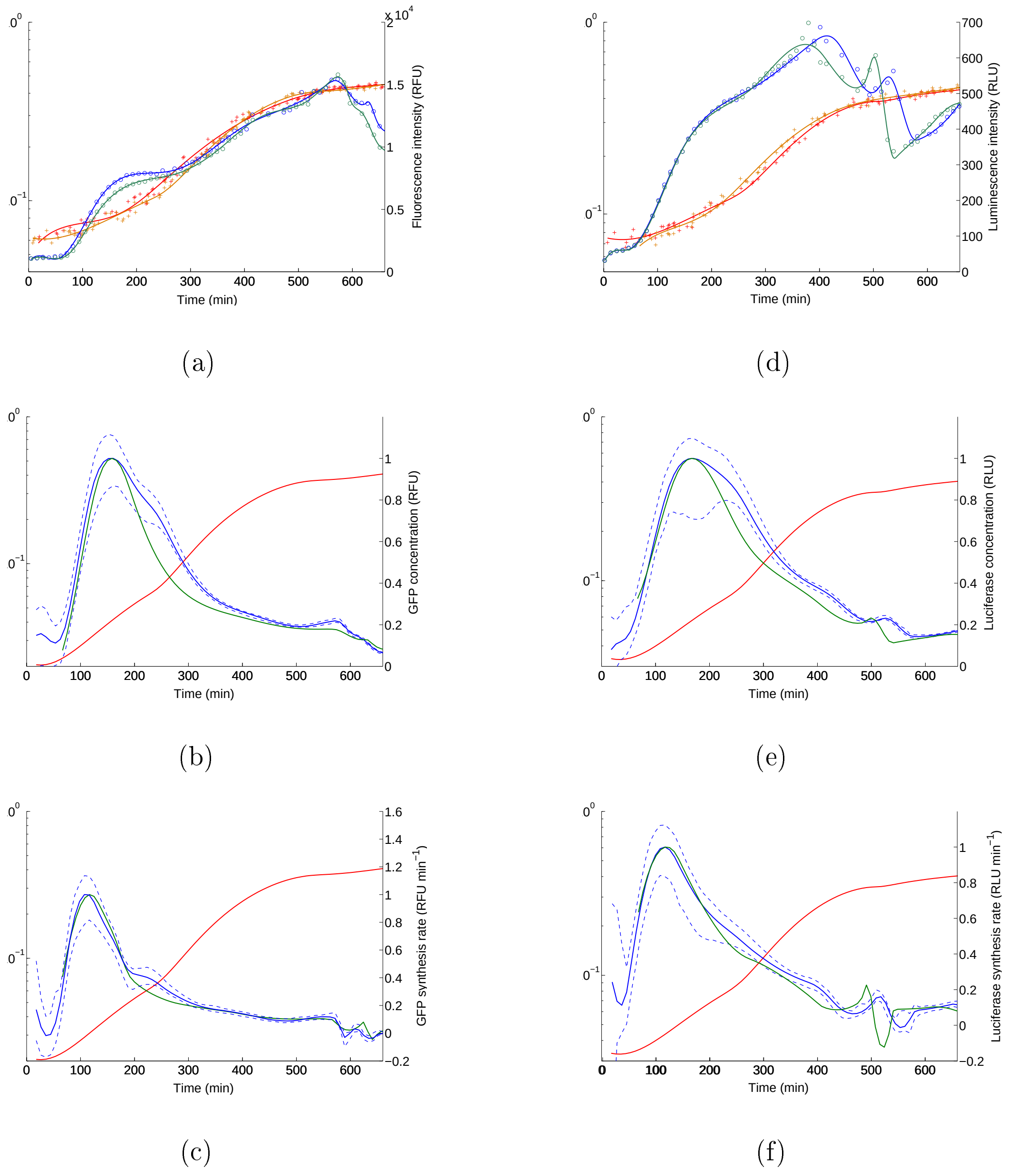

Figure 10: Reproducibility of measurements. (a) Absorbance and fluorescence measurements taken from Figure 2 in the main text, together with data from a replicate experiment. (b) GFP concentration (taken from Figure 3 in the main text), together with the GFP concentration computed from the same replicate time-series. (c) GFP synthesis rate (taken from Figure 4 in the main text), together with the synthesis rate computed from the same replicate time-series. (d)-(f) Idem for luciferase reporter data. The dashed lines denote the 95% confidence bands from the figures in the main text.

$$\frac{dm(t)}{dt} = \kappa_m\, f(t) - (\mu(t) + \gamma_m)\, m(t) \tag{S3}$$

$$\frac{dp(t)}{dt} = \kappa_p\, m(t) - (\mu(t) + \gamma_p)\, p(t) \tag{S4}$$

$$\tag{S5}$$

for the expression of the reporter gene:

$$\frac{dn(t)}{dt} = \kappa_m\, f(t) - (\mu(t) + \gamma_n)\, n(t) \tag{S6}$$

$$\frac{dq(t)}{dt} = \kappa_p\, n(t) - (\mu(t) + \gamma_q)\, q(t) \tag{S7}$$

$$\frac{dr(t)}{dt} = \kappa_r\, (q(t) - r(t)) - (\mu(t) + \gamma_q)\, r(t) \tag{S8}$$

and for the growth rate:

$$\mu(t) = \frac{d \ln A(t)}{dt} = \frac{dA(t)}{dt}\,\frac{1}{A(t)}. \tag{S9}$$

S4.1 Computation of host protein synthesis rate $\kappa_p\, m(t)$

The following differential equation can be derived from (S3):

$$\frac{d\kappa_p\, m(t)}{dt} = g(t) - (\mu(t) + \gamma_m)\, \kappa_p\, m(t), \tag{S10}$$

where $g(t) = \kappa_p\, \kappa_m\, f(t)$. The solution is a standard result from the theory of differential equations (Chapter XI in (4)):

$$\kappa_p\, m(t) = \exp^{-\int_0^t (\mu(\tau)+\gamma_m) d\tau} \left(\int_0^t g(\tau)\, \exp^{\int_0^\tau (\mu(\sigma)+\gamma_m) d\sigma}\, d\tau + \kappa_p\, m(0) \right). \tag{S11}$$

The integral in the first exponential in the right-hand side is straightforward to compute as $\int_0^t (\mu(\tau) + \gamma_m)\, d\tau) = \ln(A(t)/A(0)) + \gamma_m\, t$ by the definition (S9) of the growth rate. The integral involving the term $g(\tau)$ has to be numerically solved though. In order to achieve this, we first estimate the latter term from the primary data in the following way. After multiplying by κ_p, we obtain from (S6) that

$$g(t) = \frac{d\kappa_p\, n(t)}{dt} + (\mu(t) + \gamma_n)\, \kappa_p\, n(t). \tag{S12}$$

$\kappa_p\, n(t)$ is the reporter synthesis rate that was computed from the data as described in Section 3.3 of the main text, so that $g(t)$ can be obtained from (S12).

The actual computation of (S11) also requires the initial synthesis rate of the host protein, $\kappa_p\, m(0)$, to be known. Given that the degradation constants of the reporter and host mRNA are generally not the same, the initial mRNA concentrations will be different too (*i.e.*, $m(0) \neq n(0)$). In order to estimate the value of $m(0)$, we remind that in the experimental conditions

of this paper, the bacteria have been in stationary phase for several hours before dilution in fresh growth medium. As a consequence, just before the nutrient upshift, the concentrations of both the host and reporter protein can be assumed to have attained a steady state (Section 3.5 of the main text):

$$\frac{dm(0)}{dt} = \frac{dn(0)}{dt} = 0. \tag{S13}$$

By means of (S3), and the fact that in stationary phase the growth rate is negligible, we obtain the following expression for the initial concentration of the host mRNA

$$m(0) = \frac{\mu(0) + \gamma_n}{\mu(0) + \gamma_m}\, n(0) \approx \frac{\gamma_n}{\gamma_m}\, n(0) \tag{S14}$$

As a consistency test, it is not difficult to check that if $\gamma_m = \gamma_n$, that is, if the host and reporter mRNA have the same degradation constant, the time evolution of the two mRNA concentrations is exactly equal, as expected. In fact, it then follows from (S11) and (S14) that

$$\kappa_p\, m(t) = \exp^{-\int_0^t (\mu(\tau)+\gamma_n) d\tau} \left(\int_0^t g(\tau)\, \exp^{\int_0^\tau (\mu(\sigma)+\gamma_n) d\sigma}\, d\tau + \kappa_p\, n(0) \right) = \kappa_p\, n(t), \tag{S15}$$

and thus $m(t) = n(t)$.

S4.2 Computation of host protein concentration $p(t)$

We solve the differential equation (S4) as above:

$$p(t) = \exp^{-\int_0^t (\mu(\tau)+\gamma_p) d\tau} \left(\int_0^t \kappa_p\, m(\tau)\, \exp^{\int_0^\tau (\mu(\sigma)+\gamma_p) d\sigma}\, d\tau + p(0) \right). \tag{S16}$$

The computation involves an integral with the term $\kappa_p\, m(\tau)$, which is exactly the protein synthesis rate obtained from (S11). Notice that the computation of the latter term already implies a correction for the difference in mRNA degradation constants (γ_m and γ_n). Equation (S16) adds a correction for the difference in protein degradation constants (γ_p and γ_q). The value of $p(0)$ can be computed analogously to the value of $m(0)$, using the fact that the bacteria are in steady state and that the growth rate is negligible in stationary phase. From

$$\frac{dp(0)}{dt} = \frac{dq(0)}{dt} = 0. \tag{S17}$$

it follows with (S14) that

$$p(0) = \frac{\kappa_p\,(m(0) - n(0)) - (\mu(0) + \gamma_q)}{(\mu(0) + \gamma_p)}\, q(0) \approx \frac{\left(\frac{\gamma_n}{\gamma_m} - 1\right) \kappa_p\, n(0) - \gamma_q}{\gamma_p}\, q(0). \tag{S18}$$

Again, it is easy to verify (and therefore not shown) that $p(t) = q(t)$, if $\gamma_m = \gamma_n$ and $\gamma_p = \gamma_q$, that is, if the host and reporter gene system have the same kinetic properties.

S5 Computation of confidence intervals by means of bootstrapping

Let $\hat{f}(t)$ be the regression spline estimate of a function $f(t)$ to be recovered from the time-series data, as explained in Section S2. Moreover, let π be a mathematical transformation of $\hat{f}(t)$ yielding $\hat{q}(t)$, *i.e.* $\hat{q}(t) = \pi(\hat{f}(t))$. $\hat{q}(t)$ is an estimate of an unknown quantity $q(t)$, for instance the reporter concentration or synthesis rate (Section 3.3 of the main text).

In order to obtain an estimate of the unknown sampling distribution of $q(t)$, we follow a simple bootstrap method called residual resampling (5). Residual resampling assumes i.i.d., but not necessarily normal errors, consistent with the model in Section S2. We first compute the residuals of the optimal spline fit to the primary data at the time-points t_j, $j \in \{1, \ldots, n\}$:

$$e_j = z_j - f(t_j).$$

We then randomly resample the residuals with replacement, so as to obtain m sets of n residuals each: $\{e_j^{(k)}\}$, $k \in \{1, \ldots, m\}$. m is usually a high number (in our case good results were obtained for $m = 200$). We construct m new data sets of n measurements each, by adding the resampled residuals to the original estimate of f. That is, the jth measurement in the kth data set is given by

$$z_j^{(k)} = f(t_j) + e_j^{(k)}.$$

We then compute new optimal spline fits $\hat{f}^{(k)}$ and derived quantities $\hat{q}^{(k)}$ from the resampled data. This results in an empirically determined estimate of the sampling distribution of $q(t)$.

Several different procedures are described in the literature to infer 95% confidence intervals from the bootstrap distribution (5). For our purpose, the simple bootstrap-percentile approach turned out to be sufficient. From the bootstrap distribution, we compute the 2.5th and 97.5th percentile, labelled $\hat{q}_{0.025}(t)$ and $\hat{q}_{0.975}(t)$, respectively. The 95% confidence interval is then given by

$$[\hat{q}(t) - (\hat{q}_{0.975}(t) - \hat{q}(t)), \hat{q}(t) - (\hat{q}_{0.025}(t) - \hat{q}(t))].$$

The confidence band for $q(t)$ over the time-interval of the experiment is obtained by connecting the point-wise 95% confidence intervals at evenly-spaced time-points (80 in our case).

S6 Experimental material

The plasmids used in this study and the probes used for the Northern blots are listed in the following tables.

S7 Robustness of results for very large differences in mRNA half-life

Figure 11 extends the robustness results in the main text to the case of very large differences in the half-lives of host and reporter mRNA, by allowing the degradation constants to vary over two orders of magnitude (γ_n/γ_m equal to 0.1 and 10). As can be seen in panels (a)-(b), changing the degradation constant to $\gamma_n/\gamma_m = .1$ (orange curve) and $\gamma_n/\gamma_m = 10$ (green

Plasmid	Characteristics	Reference
pZE1RM	*gfp*-containing plasmid	(6)
pSB377	*lux*-containing plasmid	(7)
pZEgfp	pZE1RM with modified multiple cloning site	This study
pSBluc	pSB377 with modified multiple cloning site	This study
pZE-fisgfp	*fis* promoter cloned upstream of *gfp* in pZEgfp	This study
pSB-fislux	*fis* promoter cloned upstream of *lux* in pSBluc	This study
pZACR101	*lux* operon cloned downstream of the inducible PLtetO-1 promoter	Ranquet *et al.*, in preparation
pZACR105	*gfp* gene cloned downstream of the inducible $PL_{\mathrm{tetO\text{-}1}}$ promoter	Ranquet *et al.*, in preparation

Table S1: Plasmids used in this study.

Sequence	Name
5'-TGCTGTTACATATTTCCGAGGTCCG-3'	LuxB-N
5'-ATCTTCAATGTTGTGTCTAATTTTGAAG-3'	GFP-N
5'-CGCGTGTACGGCCATGAATGGTCAGAGCCTGAATGCCACAGTCTTCAGCC-3'	DusB-N

Table S2: Probes used for Northern blots.

curve) has only a moderate effect on the relative protein synthesis rate (mRNA concentration). The effect on the relative protein concentrations also remains small (panels (c) and (d)).

References

[1] Lee T: **On algorithms for ordinary least square regression spline fitting: A comparative study**. *J. Stat. Comput. Simul.* 2002, **72**(8):647–663.

[2] Hastie T, Tibshirani R: *Generalized Additive Models.* Boca Raton, FL: CRC Press 1999.

[3] Ruppert D, Wand M, Carroll R: *Semiparametric Regression.* Cambridge: Cambridge University Press 2003.

[4] Courant R: *Differential and Integral Calculus,* 2 vols. London: Blackie & Son 1959.

[5] Hamilton L: *Regression with Graphics : A Second Course in Applied Statistics.* Belmond, CA: Duxbury Press 1992.

[6] Elowitz M, Leibler S: **A synthetic oscillatory network of transcriptional regulators**. *Nature* 2000, **403**(6767):335–338.

[7] Déthiollaz S, Eichenberger P, Geiselmann J: **Influence of DNA geometry on transcriptional activation in *Escherichia coli***. *EMBO J.* 1996, **15**(19):5449–5458.

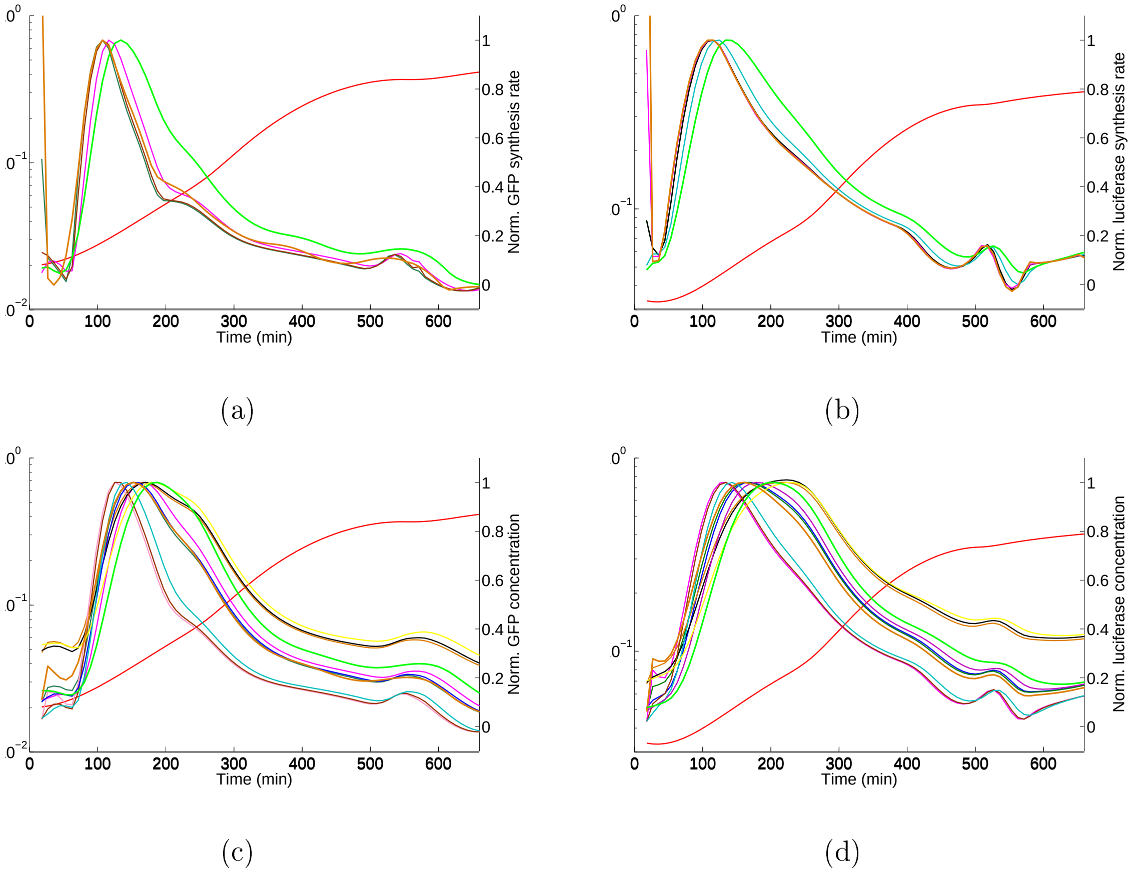

Figure 11: Correction of protein synthesis rates and concentrations for large differences in mRNA half-lives. (a) Robustness of computed protein synthesis rate (mRNA concentration) to systematic errors caused by differences in half-lives of *gfp* and *fis* mRNA. In comparison with Fig. 6 in the main text, curves for γ_n/γ_m values equal to 0.1 and 10 are shown. (b) Idem for *lux*. (c) Robustness of computed protein concentration to systematic errors caused by differences in half-lives of the products of *gfp* and *fis*. In comparison with Fig. 7 in the main text, curves for γ_n/γ_m values equal to 0.1 and 10 are shown, for the case $\gamma_q/\gamma_p = 1$. (d) Idem for *lux*.
